# Building stronger bonds: The impact of family support and communication on suicidal behaviors among Black men who have sex with men

**DOI:** 10.1111/sltb.13072

**Published:** 2024-03-15

**Authors:** Donte T. Boyd, Camille R. Quinn, Kristian V. Jones, Bernadine Waller, Evelyn Joy Coker, Erinn B. Duprey, Catherine Cerulli, Henrika McCoy

**Affiliations:** ^1^ College of Social Work, The Ohio State University Columbus Ohio USA; ^2^ Center for Interdisciplinary Research on AIDS Yale University School of Public Health New Haven Connecticut USA; ^3^ School of Social Work, University of Michigan Ann Arbor Michigan USA; ^4^ Center for Equitable Family and Community Well‐being, School of Social Work, University of Michigan Ann Arbor Michigan USA; ^5^ School of Social Work University of Washington Seattle Washington USA; ^6^ Department of Psychiatry Columbia University Irving Medical Center/New York State Psychiatric Institute New York City New York USA; ^7^ Sandra Rosenbaum School of Social Work, University of Wisconsin‐Madison Madison Wisconsin USA; ^8^ Mt. Hope Family Center Rochester New York USA; ^9^ Children's Institute Rochester New York USA; ^10^ Department of Psychiatry University of Rochester Rochester New York USA; ^11^ Community Engagement Core TRANSFORM Center, Mt. Hope Family Center Rochester New York USA; ^12^ Steve Hicks School of Social Work University of Texas at Austin Austin Texas USA; ^13^ Texas Center for Equity Promotion, College of Education, The University of Texas at Austin Austin Texas USA

**Keywords:** Black gay and bisexual males, families, suicidal behaviors

## Abstract

**Introduction:**

It has been well documented that men who identify with a sexual orientation other than heterosexual are at a greater risk for suicide‐related outcomes. What is less known are the protective factors that can reduce such negative outcomes and contribute to their resilience.

**Methods:**

This study used data collected between December 1, 2021, and January 2022 to understand how family factors contribute to or prevent depression symptoms and suicide outcomes among young Black men who have sex with men (BMSM) ages 18 to 29 (*N* = 400). A path analysis was conducted to explore the direct and indirect effects of suicide attempts.

**Results:**

Surprisingly, there were nuanced findings that showed having a family member or friend die by suicide was indirectly associated with suicide planning and suicide attempts. It was also unexpectedly noted that there was a positive relationship between higher rates of depressive symptoms and higher levels of support from family members.

**Conclusions:**

The population focused on in this study is understudied and has unique needs. Identifying familial support may not automatically reduce the thoughts and plans of young BMSM, which is an example of why their intersecting marginalized identities must be considered when conducting further research, creating interventions, and providing therapeutic services.

## INTRODUCTION

Research highlights that men who have sex with men (MSM) or individuals who identify as having a sexual orientation other than heterosexual (Suen et al., 2020) face a higher risk of suicide‐related outcomes compared to their heterosexual peers (Ramchand et al., 2022). Within these communities, MSM are likely to encounter environmental stressors such as identity‐based discrimination, financial insecurity, and social isolation, which present unique challenges to their overall well‐being (Schuler et al., 2021). Moreover, overarching oppressive systems of power impact the environments Black MSM navigate daily, potentially subjecting them to distinct or compounded experiences of stigmatization when their sexual orientation intersects with other identities such as race, class, and gender (Bowleg, 2023; Braveman & Parker Dominguez, 2021; Crawford et al., 2002; Crenshaw, 1989; Cyrus, 2017). Black men in the United States (U.S.) who identify as gay or bisexual face additional negative social exposures related to individual social identities and challenges when integrating multiple social identities (Bowleg, 2012). For example, they tend to be disproportionately impacted by adverse psychosocial and physical health outcomes such as HIV/AIDS and other sexually transmitted infections, poor mental health, and poverty (Millett et al., 2007, 2012; Wilson et al., 2016). The stress levels of equity‐deserving populations of color tend to manifest through both interpersonal and structural mechanisms due to their exposure to race‐ and sexual orientation‐based stigma, as well as limited levels of individual and social resources that may be used to alleviate stress (Hatzenbuehler, 2010; Meyer, 2010). Additionally, they can create contextual stressors and promote adverse environments jeopardizing the health of young Black MSM (Wilson et al., 2016). Exposures leading to negative mental health outcomes include racism in white LGBTQ+ spaces, negative stereotypes specific to Black MSM, and heterosexism within Black communities (Bowleg, 2012). Crenshaw (1991) suggests the experiences of marginalized people exist in multiple forms of interlocking aspects of social oppressions and the toll they exert on people of color. Specifically, she coined intersectionality to emphasize these intersecting identities and the cumulative effects they may have on the health of Black, gay, and bisexual men (Burgess‐Proctor, 2006; Owen et al., 2017). Although this theoretical framework was initially developed with women in mind, we find it relevant to the young Black MSM represented in our study. Moreover, the challenges from the individual to societal level that Black MSM experience contribute to a higher risk of suicide‐related outcomes compared to their heterosexual peers (Kelly, Drazdowski, et al., 2021).

Suicidality encompasses a range of suicidal behaviors, including ideations, planning, and attempts that may lead to suicide (Ruutel et al., 2017). Research on suicide‐related outcomes among Black MSM has mainly concentrated on identifying unique forms and rates of risk within this demographic rather than protective factors. One notable protective factor against suicide among Black MSM, studied to some extent, is the presence of familial support (Boyd, Threats, et al., 2020; Latkin et al., 2017).

### Familial factors' impact on suicidality among Black MSM

Parental constructs hold importance for individuals across various age brackets; however, they carry specific cultural and social significance for youth and young adults who have experienced adversity (Quinn et al., 2021). Boyd, Jones, et al. (2022) report that family factors related to sexual identity play a salient role in the lives of sexual minority group members. They define such factors as family support, values, and embarrassment as perceived by a person's biological parent, caregivers, and/or siblings. Prior literature has indicated that family‐level factors are essential to consider when assessing suicidal outcomes (Boyd, et al., 2022; Lindsey et al., 2019). Suicidal ideation involves contemplating suicide without necessarily making plans for it, while suicide planning usually entails devising a specific plan to carry it out (Miranda‐Mendizabal et al., 2019; Uddin et al., 2019). Quinn et al. (2021) noted that among 190 African American youth and young adults living in a mid‐Atlantic city public housing development, males were significantly more likely than females to have devised a plan to die by suicide, especially if their mothers had an alcohol problem or their fathers were incarcerated. In a study involving African American transgender individuals, findings indicate that among the 20% who have considered attempting suicide, factors such as age, marital status, income, experiences of victimization, and disclosure of transgender identity to friends and family were significantly linked to making plans for suicide (Andrew Yockey et al., 2020). However, suicide attempts differ from deaths in that they involve actions that do not result in the person's death despite the attempt. Past research has also found that family support is a protective factor against suicidal behaviors among young people (Sheftall et al., 2022). A study focused on Black college students indicated that family support was associated with a lower risk of suicidal attempts (Lincoln et al., 2012). Another study among Black and Latino youth suggested that those who reported suicide attempts were more likely to report not having access to a family network (Perkins & Hartless, 2002).

One's chosen family, which mimics nuclear family structures (Hailey et al., 2020), can also influence family factors. The “coming out” process is one of the most direct ways in which family factors can impact the sexual identity of Black MSM. During this process, family member support, or lack thereof, for the person disclosing their sexual identity is critical to the mental health of the sexual minority member. Strong family support is identified as a protective factor for gay men (Latkin et al., 2017).

Conversely, rejection by one's family can have formidable effects, impacting lives and safety. Young lesbian, gay, and bisexual individuals who come from households supportive of their sexuality experience a considerably lower risk of depression and suicidality compared to those from families with a high level of rejection (Ryan et al., 2009). Scholars suggest that demographic factors, including race, ethnicity, and socioeconomic status, can also demonstrate how interlocking aspects of social oppressions, such as heterosexism, racism, sexism, and classism at the structural level, influence the process of coming out and living as a gay or bisexual man (Bird et al., 2017; Bowleg et al., 2013). Family support can serve as a protective factor and a crucial source of assistance for Black MSM individuals; however, homophobia within the family can impede living authentically (Pastrana Jr., 2016). This challenge may lead to common indicators of suicide risk, such as increased depression, substance misuse, and suicidal thoughts and behaviors. Previous studies examining suicidal deaths among Black/African American youth and young adults highlight a connection between prior suicidal ideation, planning, and related behaviors, underscoring the prevalence and urgency of addressing suicidality within this demographic (Quinn et al., 2021). Therefore, further research in this domain is warranted.

### Ecodevelopmental framework

Ecodevelopmental theory has been employed in previous studies with male youth of color to examine and explain correlates of substance misuse and HIV testing (Boyd, Threats, et al., 2020; Cordova et al., 2018, 2020). Drawing on elements of social ecological theory, developmental theory, and a focus on social interactions, ecodevelopmental theory comprises four interrelated systems: microsystem, mesosystem, ecosystem, and macrosystem (Bronfenbrenner, 1979; Szapocznik & Coatsworth, 1999). The microsystem includes families, peers, school, and neighborhood systems, while the mesosystem represents the connections between two or more microsystems, such as the intersections among youth, their families, and their friends.

Notably, ecodevelopmental theory emphasizes the role of family functioning and interactions among risk and protective processes from a developmental perspective (Nathanson et al., 1986). In this context, we focus on Black males to conceptualize how family factors and highly influential microsystems in their lives impact suicidal outcomes for Black MSM. The ecodevelopmental theoretical framework examines factors that can affect suicide among Black MSM, which is crucial given the limited literature in this area. Additionally, considering the importance of family factors and the specific risks for adverse mental health outcomes for young Black MSM, more research is needed to understand how family factors could potentially mitigate or exacerbate suicide outcomes for this specific demographic.

### Current study

Black men are an understudied group in suicide research (Bennett Jr & Joe, 2015; Joe et al., 2018). Moreover, existing research does not reflect the nuances of within‐group differences and tends to focus on homogeneous outcomes (Goodwill et al., 2021). Very few studies focus on young Black MSM, despite their higher risk for suicide outcomes than their heterosexual peers (Kelly, Drazdowski, et al., 2021; Ramchand et al., 2022). Consequently, this study utilizes ecodevelopmental theory (Boyd, et al., 2022; Cordova et al., 2016) to examine how familial support contributes to or prevents suicide outcomes among young Black MSM ages 18–29.

Due to ecodevelopmental theory's focus on both risk and protective factors in one's environment, we utilize it to assess the protective impact of family factors on suicide outcomes among young Black MSM. We extend the theory's utility by examining the effect of family factors on depression symptoms, suicidal thoughts, suicide planning, and suicide attempts. We propose five hypotheses: (a) Strong family bonds correlate with reduced depression and lower suicidal thoughts; (b) Increased open family communication is linked to lower depression and decreased risk of suicidal thoughts; (c) Experiencing the suicide of a family member or friend is associated with heightened depression symptoms and suicidal thoughts; (d) Elevated depression symptoms and suicidal thoughts are connected to suicide planning; (e) Family bonding and open communication indirectly relate to lower depression, suicidal thoughts, planning, and attempts.

## METHODS

### Study procedures and recruitment

The survey utilized Qualtrics software for data collection, recruiting participants through various channels, including social media sites (Facebook and Twitter), Amazon Mechanical Turk (MTurk), and community‐based organizations (Beymer et al., 2018; Boyd et al., 2023). To enlist participants, an anonymous survey link was generated and featured on a recruitment flyer. This flyer was distributed through Facebook, Twitter, and MTurk by the principal investigator (PI) and research assistants every morning at 8 a.m. Eastern Standard Time. Additionally, the PI and research assistants shared the flyer with LGBTQI+ youth and young adult‐focused community‐based organizations across the U.S., who then disseminated the anonymous link to eligible participants.

MTurk was employed as a cost‐effective and efficient method of recruiting participants from various disciplines, including public health research (Pacek et al., 2019). To be eligible for the survey, individuals registered with MTurk needed to have a previous survey approval rating of 95% or higher, be at least 18 years old, and reside in the U.S. These criteria were verified during the initial MTurk registration process (Pacek et al., 2019; Rass et al., 2015; Walters et al., 2018). Upon logging into the MTurk platform during the survey week, potential respondents were informed about the opportunity to take a survey on HIV and assets for Black MSM. They were advised that the survey would take approximately 20 min, new surveys would be released every morning at 8 a.m. Eastern Standard Time, and participants were instructed to complete the survey in one sitting. Compensation included one dollar, along with other incentives provided by MTurk (Pacek et al., 2019; Walters et al., 2018).

For community‐based organizations, the research team shared the flyer with community health workers, including an anonymous survey link. These health workers then distributed the flyer to eligible participants who were considered clients of their organization. Recruitment occurred on December 1, 2021, and January 31, 2022. Individuals who completed the 20‐min survey and provided an email address received a $35 electronic Amazon gift card.

To ensure data quality and prevent fraudulent responses, our survey implemented measures provided by Qualtrics Survey Protection. The research team monitored IP addresses to verify that respondents were located within the U.S., thus maintaining data integrity and preventing multiple responses from the same participant. Speeding checks were also employed, identifying respondents with a survey duration less than or equal to one‐third of the median survey duration and excluding them from the final sample. The utilization of Qualtrics survey protection included additional tools to prevent manipulative tactics, such as ballot box stuffing (which places a cookie in the browser after a response is submitted), reCAPTCHA scores (which asks respondents to identify particular items in pictures or replicate a series of letters before starting the survey), and bot detection (using a Qualtrics survey question that indicates the likelihood of a respondent being a bot based on reCAPTCHA scores). These measures were implemented to uphold data quality and ensure the authenticity of participant responses.

### Participants

The inclusion and exclusion criteria were consistent across all sampling sites. To participate in the study, individuals had to self‐identify as Black or African American, be between the ages of 18 and 29, reside in the U.S., be assigned male at birth, be fluent in English, currently identify as a man, and report engaging in sexual contact (oral, anal, or otherwise) with a male within the past year. Respondents who did not meet these criteria were immediately redirected out of the survey. To ensure that all participants answered each question, we utilized the forced response option in Qualtrics, requiring participants to provide a response for every question. This approach enabled us to gather complete and consistent data from all respondents.

Upon clicking on the survey link, participants were presented with informed consent and asked to complete a screening tool to assess study eligibility. Those who met the inclusion criteria were then asked a series of questions on demographics, developmental assets, mental health, and other protective mechanisms. Participants using social media sites and MTurk to complete the survey utilized their own computers. Those completing the survey in a community‐based organization used a computer or tablet provided by the organization. The study received approval from The Ohio State University Institutional Review Board (IRB #2021E1175).

The final sample comprised 400 participants identifying as Black MSM aged 18 to 29. The average age was 23.46 years, with a standard deviation of 2.59. The majority (*n* = 200) were recruited from MTurk, with 100 from community‐based organizations and another 100 from social media sites. Racial/ethnic identity distribution was 75% Black American or African American, 10% Caribbean, 10% Afro‐Latino, and 5% continental African. Regarding education, 28% had not attended high school, while 29% had completed college or post‐graduate education. Household income ranged from less than $20,000 to $150,000, with an average of $57,499. Notably, 95% reported being assigned male at birth, while 5% identified as trans men.

The research team actively sought to include and encourage participation from trans men by incorporating specific information on flyers and other recruitment materials. This initiative aimed to ensure representation and collect comprehensive data on the experiences of this population. All participants self‐reported having had sex with men within the last year. Regarding sexual orientation, 45% identified as gay, 35% as straight or heterosexual, 10% as bisexual, 5% were questioning their sexual orientation, and 5% identified as other.

## MEASURES

### Outcome variable: Past year suicide attempt

Suicide attempts were assessed using a single item that asked respondents to indicate whether they had attempted suicide within the previous 12 months. Response categories were 1 = *yes* and 0 = *no* (Goodwill et al., 2021). Twenty‐seven percent of participants reported attempting suicide in the past year.

### Mediator variables

#### Past‐year suicide planning

Suicide attempts were assessed using a single item that asked respondents to indicate whether they had attempted suicide within the previous 12 months. Response categories were 1 = yes and 0 = no (Goodwill et al., 2021). Twenty‐seven percent of participants reported attempting suicide in the past year.

#### Suicidal ideation

Suicidal ideation was measured using a single item that asked participants, “Have you seriously considered ending your life within the past 12 months?” Response categories were 1 = *yes* and 0 = *no* (Goodwill et al., 2021). Thirty‐eight percent of participants had seriously considered ending their lives in the past 12 months.

#### Depression symptoms

We utilized the Center for Epidemiological Studies Depression Scale (CESD‐10) to measure depression symptoms (Wheeler et al., 2019). The CESD‐10 assesses depressive symptoms experienced in the past week. Previous research has validated the measure among clinically depressed populations, the general population, and sexual minorities of color (English et al., 2020). Sample items included “How many times in the past week did you feel as good as other people?” and “How many times in the past week did you have trouble keeping your mind on task?” Response options ranged from 0 (*Rarely or never*) to 3 (*Most or all of the time*). The CESD‐10 scores range from 0 to 30, with higher scores indicating more depressive symptoms (Cronbach's *α* = 0.81). Individuals with scores above 20 were classified as having moderate to severe depression symptoms (Andresen et al., 1994; Okafor et al., 2020).

### Independent variables

Parental support was assessed using three items on a 5‐point Likert scale ranging from 1 (*strongly disagree*) to 5 (*strongly agree*). Participants responded to statements such as “My parents give me help and support when I need it.” The average of responses to these three items was calculated, with higher scores indicating more family support (Toomey et al., 2019). The Cronbach's alpha for this measure is 0.95.

Open family communication was assessed using a single item on a 5‐point Likert scale ranging from 1 (*Strongly disagree*) to 5 (*Strongly agree*). Participants were asked to rate the statement “I have lots of good conversations with my parents” (Syvertsen et al., 2021).

Participants provided their response regarding family or friend suicide using a single item that asked, “Have any of your family members or friends died by suicide in the past 12 months?” Response categories were 1 = *yes* and 0 = *no*. Thirty‐one percent of participants reported having a family member or friend who had died by suicide.

### Statistical analysis

Our team employed Mplus version 8.7 to conduct a path analysis, and there was no missing data. As a first step, the distribution of all study variables was checked. Participant characteristics are presented in Table 1, while Table 2 displays correlations among study variables. In Table 3, we examined whether family factors (perceived parent support, open family communication, and having a family member or friend die by suicide) were associated with depression symptoms and suicidal thoughts (Muthén & Muthén, 2017). Path analysis enabled the testing of the indirect effects of family factors on suicide planning and attempts. We utilized the means and variance‐adjusted weighted least squares estimator instead of maximum likelihood estimation, as this estimator is preferred when the dependent variable is categorical and the data are not normally distributed (Kline, 2015). Model fit indexes were not provided in the study as the model was just identified (Kline, 2015). Instead, standardized beta coefficients and *p*‐values were included to examine associations among the study variables.

**TABLE 1:** Demographics and sample characteristics (*N* = 400).

Variable	*M* or %	SD or *N*	Range (if applicable)
Age	23.46	2.59	18–29
Household income	$57,499.50	1.34	Up to $150,000
Race and ethnicity			
Black American or African American	75%	300	
Caribbean (e.g., Jamaican, Haitian)	10%	40	
Continental African (e.g., Nigerian, Ghanian)	5%	20	
Afro‐Latino (e.g., Dominican)	10%	40	
Sex assigned at birth			
Male	95%	380	
Female	5%	20	
Sexual orientation			
Heterosexual or straight	35%	140	
Gay	45%	180	
Bisexual	10%	40	
Questioning	5%	20	
Other	5%	20	
Education			
Never attended school	30%	100	
Less than high school	19%	65	
Some high school	4%	12	
High school diploma or GED	2.4%	8	
Some college, associate degree	14%	47	
College, postgraduate	29%	98	
Currently in school	1.5%	5	
Depressive symptoms	14.46%	5.97	
Laid off due to COVID 19			
Yes	83%	250	
No, I was laid off for other reasons	14%	50	
Not applicable, I was not working prior	14%	50	
Parent Support	5.17	1.52	1–5
Open family communication	3.88	1.03	1–5
Suicide planning			
Yes	34%	130	
No	66%	220	
Suicide attempt			
Yes	28%	98	
No	72%	252	
Suicidal thoughts			
Yes	38%	128	
No	62%	206	
Suicide of family or friend			
Yes	31%	101	
No	69%	224	

**TABLE 2:** Correlation of key study variables (*N* = 400).

Variable	1	2	3	4	5	6
1. Suicide attempts	—					
2. Suicide planning	0.62***	—				
3. Suicidal thoughts	0.67***	0.74***	—			
4. Depression symptoms	0.40***	0.45***	0.51***	—		
5. Parental support	−0.07	−0.06	0.01	0.04	—	
6. Open family communication	0.01	0.09	0.05	0.09	0.11*	—
7. Suicide of family member or friend	0.64***	0.65***	0.61***	0.44***	−0.06***	0.05

*Note*: *p < 0.05, ***p < 0.001.

**TABLE 3:** Direct effects of family factors, depression symptoms, suicidal thoughts, and behaviors (*N* = 400).

	*B*	*β*	SE
Direct effects			
Suicidal thoughts			
Depression symptoms	0.02	0.23***	0.05
Parent support	0.08	0.17**	0.05
Open family communication	−0.03	−0.07	0.04
Family member or friend's suicide	0.48	0.17***	0.04
Depression symptoms			
Parent support	2.73	0.47***	0.05
Open family communication	−1.01	−0.16***	0.05
Family member or friend's suicide	3.73	0.29**	0.04
Suicide planning			
Suicidal thoughts	0.66	0.68***	0.04
Depression symptoms	0.07	0.08	0.05
Suicide attempts			
Suicide planning	0.58	0.61***	0.04

*Note*: *p < 0.05, **p < 0.01, ***p < 0.001.

Abbreviations: *β*, standardized coefficients; *B*, unstandardized coefficients; SE, standard errors.

## RESULTS

Correlation results (Table 2) revealed that suicide planning was positively associated with suicide attempts (*r* = 0.62, *p* < 0.001). Suicidal thoughts were positively associated with suicide planning (*r* = 0.67, *p* < 0.001) and suicide attempts (*r* = 0.74, *p* < 0.001). Depression symptoms were positively associated with suicide attempts (*r* = 0.40, *p* < 0.001), suicide planning (*r* = 0.45, *p* < 0.001), and suicidal thoughts (*r* = 0.51, *p* < 0.001). A family member or friend dying by suicide was positively associated with suicide attempts (*r* = 0.64, *p* < 0.001), suicide planning (*r* = 0.65, *p* < 0.010), suicidal thoughts (*r* = 0.61, *p* < 0.001), depression symptoms (*r* = 0.44, *p* < 0.001), and perceived parental support (*r* = −0.06, *p* < 0.001).

**FIGURE 1:**
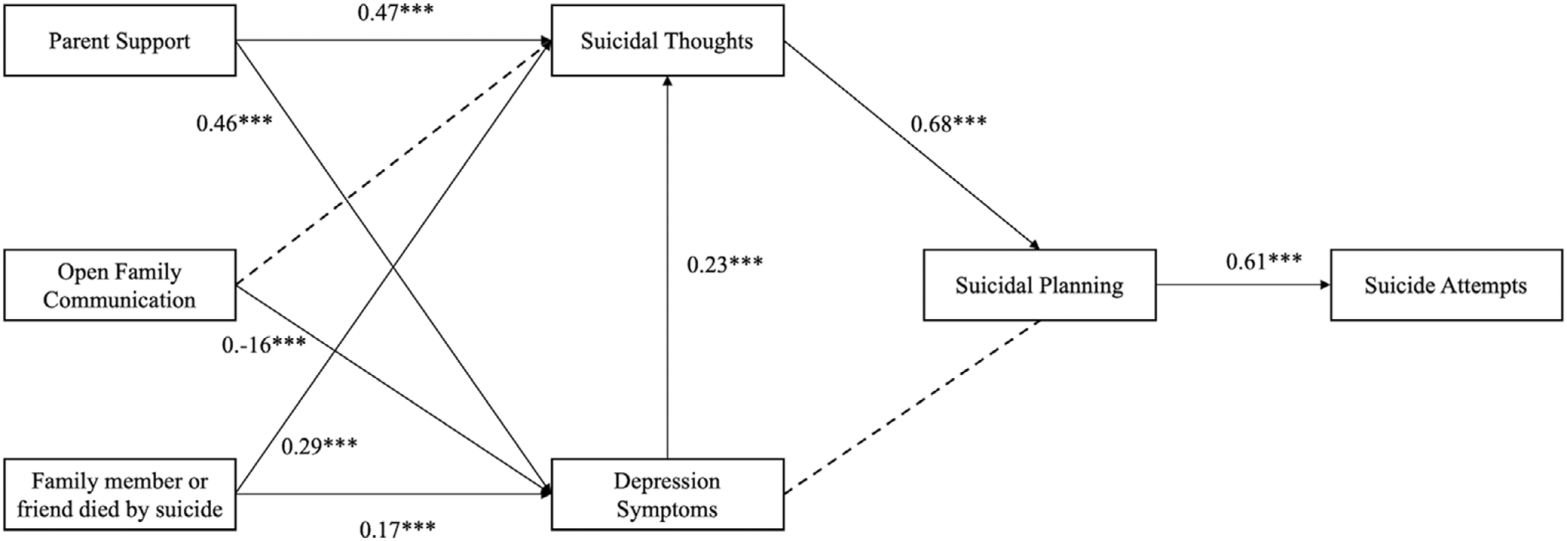
Path Model from family factors to depression symptoms and suicidal thoughts on suicidal planning and attempts (*N* = 400). *p* < 0.05*, *p* < 0.01**, *p* < 0.001***; standardized betas reported; broken lines represent non‐significant paths.

The main path model illustrates the direct effects among (1) perceived parental support, open family communication, and a family member or friend who died by suicide‐on‐suicide thoughts and depression symptoms; (2) suicidal thoughts and depression symptoms on suicide planning; and (3) suicide planning on suicide attempts (refer to Table 3 and Figure 1). Depression symptoms were positively associated with suicidal thoughts (*β* = 0.23, *p* < 0.001).

Parental support was also positively associated with depression symptoms (*β* = 0.47, *p* < 0.001) and suicidal thoughts (*β* = 0.17, *p* < 0.001). Open family communication showed a direct and negative association with depression symptoms (*β* = −0.16, *p* < 0.001). Having a family member or friend who died by suicide was positively associated with suicidal thoughts (*β* = 0.17, *p* < 0.001) and depression symptoms (*β* = 0.29, *p* < 0.01). Suicidal thoughts were positive and directly associated with suicide planning (*β* = 0.68, *p* < 0.001). Suicide planning was positively associated with suicide attempts (*β* = 0.61, *p* < 0.001).

### Indirect effects

Our results also indicated that suicidal thoughts (*B* = 0.33, *p* < 0.001) and depression symptoms (*B* = 0.01, *p* < 0.001) were both indirectly and positively associated with suicide attempts through suicide planning. Parental support (*B* = 0.06, *p* < 0.001) was indirectly and positively associated with suicide attempts through depression symptoms and suicidal thoughts.

Open family communication (*B* = −0.02, *p* < 0.001) was indirectly and negatively associated with suicide attempts via suicidal thoughts and suicide planning. Having a family member or friend who died by suicide (*B* = 0.23, *p* < 0.001) was indirectly and positively associated with suicide attempts through depression symptoms and suicidal thoughts. Parental support (*B* = 0.10, *p* < 0.001) and having a family member or friend die by suicide (*B* = 0.42, *p* < 0.001) were both positively and indirectly associated with suicide planning via suicidal thoughts and depression symptoms. Open family communication (B = −0.04, *p* < 0.001) was indirectly and negatively associated with suicide planning through depression symptoms and suicidal thoughts (refer to Table 4).

**TABLE 4:** Tests of indirect effects between family factors, depression symptoms, suicide thoughts, and behaviors.

	*B*	Standard errors	*p*
Suicide attempts			
Suicidal thoughts ➔ Suicide Planning ➔ Attempts	0.33***	0.03	0.001
Depression symptoms ➔ Suicide Planning ➔ Attempts	0.01***	0.02	0.001
Parent support ➔ Suicide thoughts ➔ Depression symptoms ➔ Suicide Planning ➔ Attempts	0.06***	0.01	0.001
Open family communication ➔ Suicide thoughts ➔ Depression symptoms➔ Suicide Planning ➔ Attempts	−0.02**	0.01	0.01
Family member or friend's suicide ➔ Suicide thoughts ➔Depression symptoms➔ Suicide Planning ➔ Attempts	0.23***	0.02	0.001
Suicide planning			
Parent support ➔ Suicide thoughts ➔Depression symptoms ➔ Planning	0.10***	0.02	0.001
Open family communication ➔ Suicide thoughts ➔ Depression symptoms ➔ Planning	−0.04***	0.02	0.001
Family member or friend's suicide ➔ Suicide thoughts ➔ Depression symptoms ➔ Planning	0.42***	0.04	0.001

*Note*: *p < 0.05, **p < 0.01, ***p < 0.001.

Abbreviations: *B*, unstandardized coefficients; *p*, *p*‐value/significance.

## DISCUSSION

The current investigation examined whether specific family factors (perceived parental support, open family communication, and experiencing a family member or friend's suicide) influence depression and suicidal thoughts and behaviors among young Black MSM aged 18 to 29 in the U.S. A path analysis was conducted to understand how various social factors impact participants' depression and suicidal thoughts and behaviors. Recognizing that depression and the quality of close relationships influence suicidal thoughts, these were explored as potential pathways for suicide‐related behaviors. Ecodevelopmental theory (Bronfenbrenner, 1979; Szapocznik & Coatsworth, 1999) was employed to assess potential linkages at the micro‐ and mesosystem levels—between parental support and open family communication, as well as depressive symptomology and suicidal behavior, including planning and attempts.

We also aimed to analyze the association of first‐degree proximal relationships with those who have died by suicide. Specifically, we examined the relationship between family and friends who have died by suicide and participants' suicide attempts. Consistent with ecodevelopmental theory (Bronfenbrenner, 1979; Szapocznik & Coatsworth, 1999), family factors (i.e., open family communication and perceived parental support) were associated with depressive symptoms and suicidal thoughts among this population. Depressive symptoms and suicidal thoughts were also directly and positively associated with suicide planning. Having a family member or friend die by suicide was indirectly associated with suicide planning and attempts (Table 3 and Figure 1). These results have clinical and policy implications for addressing suicidal thoughts and behaviors among young Black MSM.

Open family communication was directly associated with a decrease in depressive symptoms, aligning with previous research findings (Ryan et al., 2009). Additionally, family communication was indirectly tied to lower suicidal attempts via reductions in suicidal thoughts and depressive symptoms, implying nuances in communication quality. Additionally, it was linked to a decrease in suicidal planning through its influence on thoughts and depressive symptoms. Notably, no significant association was found between open family communication and suicidal thoughts. Our hypothesis posited that higher levels of open family communication would be linked to lower depressive symptoms and a reduced risk of suicidal thoughts, and we conclude that this hypothesis was partially supported. Toomey et al. (2019) conducted a study examining simultaneous associations between youths' developmental assets and suicidal behavior by sexual orientation with a sample of 116,925 U.S. students (aged 11–19). Their findings suggest that all sexual orientations other than heterosexual reported lower levels of open family communication—an external developmental asset. Open family communication was operationalized as “whether a young person communicates with parent(s) and is willing to seek advice and counsel from them,” assessed with two items, including “I have lots of good conversations with my parents” (Toomey et al., 2019, p. 791) and “If you had an important concern about drugs, alcohol, sex, or some other serious issue, would you talk to your parent(s) about it?” (Syvertsen et al., 2021; Toomey et al., 2019, p. 791). This study's sample comprised 95% of youth who identified as either heterosexual or mostly heterosexual, with over 60% being white. Consequently, when aiming to reduce depression and mitigate suicide risk among young Black men, particularly those who are gay or bisexual, it is crucial to consider specific demographic and social factors (Goodwill et al., 2021). This underscores our earlier argument that Black MSM face unique stressors and adverse social exposures related to their intersecting individual social identities (Bowleg, 2012), contributing to challenges such as adverse psychosocial and physical health issues, poor mental health, and poverty (Millett et al., 2007, 2012; Wilson et al., 2016). These findings underscore the importance of fostering open communication and providing supportive family environments to prevent suicide among Black MSM. They also suggest that strengthening family relationships and promoting healthy communication within families can contribute to better mental health outcomes and reduce the risk of suicide.

### Depression and suicidal thoughts

Consistent with our first hypothesis, all family factors—perceived parental support, open family communication, and having a family member or friend who died by suicide—were significantly associated with higher rates of depressive symptoms. Aligned with the mesosystem level of ecodevelopmental theory, young Black MSM with a family member or close friend who died by suicide face an elevated risk of experiencing depression, a known risk factor for suicide. Furthermore, the severity of depressive symptoms may place them on the trajectory of contemplating suicide. Our study builds upon a systematic review indicating specific demographic indicators as suicide risk factors, including male gender, family history of suicide, and more severe depression (Hawton et al., 2013).

The results of this study also highlight the influence relationships have on the mental well‐being of Black MSM. Young men whose family members or friends experienced premature death due to self‐injury are more likely to suffer from depression. These findings align with previous research in this area, including research on the direct effect of depressive symptoms and suicidal thoughts (refer to Figure 1) (Goodwill et al., 2021). In addition to our main findings, an unexpected and notable discovery was the positive association between perceived parent support and depression. To put it more plainly, young Black MSM reported higher rates of depressive symptoms if they also reported higher levels of support from their family members. This finding in our study contradicts the existing literature. The authors hypothesized direct associations between family factors and suicidal thoughts, with indirect associations to suicide attempts among Black MSM. Additionally, they posited that suicidal thoughts may precede suicide attempts based on prior research. The research team expected that family factors, such as open family communication and family support, would directly impact individuals' experiences of suicidal thoughts and indirectly influence the likelihood of engaging in suicide attempts. While acknowledging that suicidal thoughts may not always lead to attempts, in this study, they believed these thoughts would serve as an indicator and potential precursor to actual suicide attempts, aligning with existing research (O'Donnell et al., 2004). Exploring these hypotheses aimed to deepen understanding of the role of family dynamics in shaping the mental health and well‐being of Black MSM and inform future interventions and support strategies tailored to this population.

A robust body of scholarship indicates that social support from family members is a protective factor for young men from historically marginalized populations (Boyd, Jones, et al. (2022; Boyd, et al., 2022; Britt‐Spells et al., 2018). However, young Black MSM represent a unique and understudied population, suggesting they may require higher levels of support and may be more symptomatic, necessitating greater assistance (Quinn et al., 2020). Additionally, Black MSM may face strained relationships with their families of origin related to their sexual minority identity (Pastrana Jr., 2016). While these questions are not the focus of this study, it is essential to note the multiplicative effects of young Black MSM identities, involving heightened stress and stigma associated with their poor mental health, risk for HIV/AIDS, other, and poverty in their lives and familial relationships. This highlights the need to examine various aspects of identity with an intersectional approach to better understand this population's family relationships, mental health, and suicidal behavior (O'Brien et al., 2016; Potter, 2015; Quinn et al., 2021).

### Suicide planning

Perceived parent support and having a family member or friend who died by suicide were found to have an (indirect) significant and positive association with suicide planning. This indicates a positive indirect pathway between perceived parent support and depression. Participants who reported perceived parental support also reported higher rates of suicide planning. These pathways provide a critical empirical basis for future research exploring the links between young Black MSM and parental support, including chosen (or created) families, which is understudied. Hailey et al. (2020) conducted a systematic review of African American social networks, noting diverse protocols for interaction and dynamics that may directly positively impact health equity. However, it is essential to consider the possibility that this finding could be spurious, as unidentified variables may reduce suicidal behaviors. Considering the cross‐sectional nature of the data, it is also possible that the planning preceded the familial support and merits further exploration.

The negative and indirect relationship between open family communication and suicide planning aligns with the limited literature in this area. While some studies have included communication measures, there is a lack of focus on open family communication, specifically with young Black MSM. Toomey et al. (2019) suggest disparities in access to and endorsement of internal and external assets, such as support for open family communication. This finding is in line with prior research on external supports and supportive climates (Russell & Fish, 2016; Toomey et al., 2019). It underscores that the initial point for intervention to reduce mental health disparities among young Black MSM is through the promotion of family connections—increasing knowledge and acceptance and removing systemic barriers. This approach aligns with the mesosystem level of ecodevelopmental theory.

### Suicide attempts

Young Black MSM who experienced parental support and had a family member or friend die by suicide were more likely to report higher rates of suicide attempts indirectly via suicide planning. This result is unexpected and runs counter to past research. For instance, one study noted that bisexual youth with high rates of suicidal ideation and attempts experienced more risk factors such as depression as well as a lack of parent‐family connection when compared to primarily straight and other sexual minority youth (Horwitz et al., 2021). Our study's finding is critical to understanding the mental health context of young Black MSM in the micro‐ and mesosystem levels of ecodevelopmental theory.

### Limitations

These study findings should be interpreted in the context of several limitations. First, the study is cross‐sectional, limiting our ability to infer causality and establish temporal ordering of the study variables. Despite being cross‐sectional, recent longitudinal studies have confirmed the directionality of the effects of family bonding, mother‐and‐father communication, and health outcomes among Black youth (Boyd, Quinn, & Aquino, 2020; Boyd, Threats, et al., 2020). Another study examined the directionality of parental support and non‐suicidal self‐injury (Victor et al., 2019), while a meta‐analysis of longitudinal studies explored gender differences in suicidal behaviors among youth and young adults (Miranda‐Mendizabal et al., 2019). Given the support from prior studies for the order of variables in our model and the acknowledgment that tests of indirect effects with cross‐sectional data can still be appropriate for examining emerging theoretical relationships among variables (DeBlaere et al., 2022; Spector, 2019), our empirical and theoretically informed model seemed suitable for testing in the present study. However, it is important to note some limitations. Firstly, the measure in our study does not distinguish between friends or family, leaving uncertainty about the specific effects of family or friends. Secondly, the sample recruited in this study may differ from other populations of Black MSM who did not participate. Thirdly, due to constraints in our research design, both our measures of suicidal thoughts and behaviors and open family communication consisted of only a single item.

Finally, although not directly measured in our study, we referenced Crenshaw's intersectionality theory, which posits that individuals with multiple marginalized identities, such as young Black MSM, may have unique lived experiences. However, we did not quantify or measure this aspect of intersectionality in our research, preventing a quantitative analysis of the additive impact of oppression‐related stress. Future research should consider employing mixed‐methods approaches with young Black MSM to explore their intersectional stigma over multiple time points. Scales like the Everyday Discrimination Scale could be utilized to gain insights into how racism manifests differently for this population. Semi‐structured interviews will enable researchers to delve deeper into the intersectional stigma experienced by individuals with multiple identities, particularly those often overlooked in health research. By utilizing both quantitative and qualitative methods, our goal is to assess the accumulation of specific identity‐based stressors, such as racism, sexism, and transphobia. This comprehensive approach aims to provide a more nuanced perspective on the experiences of young Black MSM, allowing us to better understand the impacts of various forms of discrimination. Recognizing the unique challenges faced by individuals with intersecting marginalized identities will enable the development of more targeted interventions and support systems to address their specific needs.

### Clinical and policy implications

Our findings contribute valuable insights for social work clinicians, psychologists, and other mental health care providers working with young Black MSM. We highlighted various aspects of suicidal behavior—suicidal thoughts (and depression as a precursor), planning, and attempts—which underlie the significant spike in suicide rates among Black youth and young adults (Boyd, et al., 2022; Paley, 2022; Quinn et al., 2022; Sheftall et al., 2022). The observed relationship between depression severity and suicidal thoughts suggests that clinicians and practitioners can adopt a preventative approach, which could be crucial for reducing depression and suicide risk among young Black MSM.

Practitioners can enhance their effectiveness in working with young Black MSM individuals by providing confidentiality, knowledge, and respect and by exhibiting warmth. Setting goals and assigning homework to achieve them, along with ensuring therapeutic components that enhance client skills in anger management, coping, and communication, are crucial strategies. Moreover, adopting a healing‐centered approach in therapeutic conversations is recommended, especially when addressing potential traumas (Ginwright, 2018). Identifying sources of trauma early in the treatment planning process can promote engagement, offering valuable insights for clinicians as they develop and set goals to improve treatment outcomes with the client (Center for Substance Abuse Treatment [US], 2014; Quinn & Grumbach, 2015).

These findings have other important implications. The insight that familial support may not automatically reduce a participant's thoughts and plans for suicide raises questions about additional factors at play. The ecodevelopmental model considers participants' mental health and support within familial and friend networks. However, is it possible that even a supportive family cannot lessen the burden of navigating disjointed systems, the difficulty in accessing mental health care, or the structural racism embedded within mental health services? Given the scarcity of mental health providers who represent sexual, racial, and cultural minority groups or the intersection of those identities, do our participants seek less care outside the comfort of the family, contributing to their ongoing burden of suicide risk? Additional work is needed to understand the intersectionality of identity and how care can be provided in a way that is sensitive to an individual's needs as a whole person rather than just one identity.

## CONCLUSION

Youth engaging in suicidal behavior often endure intense pain, exacerbated by strained family relationships or familial histories of mental health issues such as depression. While there is some literature on suicide and depression among Black men and limited studies on depression and suicide among gay and bisexual men, there is a notable gap in scholarship addressing the family factors influencing Black MSM experiences with depression and suicide. Consequently, there is a need for a greater focus on practitioners' ability to demonstrate compassion, accurate empathy, and provide culturally appropriate care. Young Black MSM represent a unique and understudied population, underscoring the importance for clinicians to build effective communication, reduce identity‐related stigma, and offer messages of hope to promote positive change in their micro‐ and mesosystems (Bronfenbrenner, 1979; Newman, 2018; Szapocznik & Coatsworth, 1999).

## CONFLICT OF INTEREST STATEMENT

The authors have no conflicts of interest to declare. Data availability statement: the datasets generated and/or analyzed data are available from the corresponding author upon reasonable request.
